# Identification of Self-Incompatibility Alleles by Specific PCR Analysis and *S-RNase* Sequencing in Apricot

**DOI:** 10.3390/ijms19113612

**Published:** 2018-11-15

**Authors:** Sara Herrera, Javier Rodrigo, José I. Hormaza, Jorge Lora

**Affiliations:** 1 Unidad de Hortofruticultura, Centro de Investigación y Tecnología Agroalimentaria de Aragón (CITA), Instituto Agroalimentario de Aragón-IA2 (CITA-Universidad de Zaragoza), 50059 Zaragoza, Spain; sherreral@aragon.es (S.H.); jrodrigo@cita-aragon.es (J.R.); 2 Instituto de Hortofruticultura Subtropical y Mediterránea La Mayora (IHSM La Mayora-UMA-CSIC), 29750 Algarrobo-Costa, Málaga, Spain; ihormaza@eelm.csic.es

**Keywords:** apricot, Gametophytic Self-Incompatibility, pollen TUBE, pollination, *Prunus armeniaca*, *S*-alleles, *S*-genotype

## Abstract

Self-incompatibility (SI) is one of the most efficient mechanisms to promote out-crossing in plants. However, SI could be a problem for fruit production. An example is apricot (*Prunus armeniaca*), in which, as in other species of the Rosaceae, SI is determined by an S-RNase-based-Gametophytic Self-Incompatibility (GSI) system. Incompatibility relationships between cultivars can be established by an *S*-allele genotyping PCR strategy. Until recently, most of the traditional European apricot cultivars were self-compatible but several breeding programs have introduced an increasing number of new cultivars whose pollination requirements are unknown. To fill this gap, we have identified the *S*-allele of 44 apricot genotypes, of which 43 are reported here for the first time. The identification of *S_c_* in 15 genotypes suggests that those cultivars are self-compatible. In five genotypes, self-(in)compatibility was established by the observation of pollen tube growth in self-pollinated flowers, since PCR analysis could not allowed distinguishing between the *S_c_* and *S*_8_ alleles. Self-incompatible genotypes were assigned to their corresponding self-incompatibility groups. The knowledge of incompatibility relationships between apricot cultivars can be a highly valuable tool for the development of future breeding programs by selecting the appropriate parents and for efficient orchard design by planting self-compatible and inter-compatible cultivars.

## 1. Introduction

Different physical and genetic strategies have been developed by plants to prevent self-pollination and promote out-crossing, and thus, ensure genetic variability [1]. A physical barrier can be found in bisexual flowers by different temporal maturation of the male and female parts (dichogamy) or by a spatial separation of the female and male parts (herkogamy) [2]. The genetic barrier, self-incompatibility (SI), is considered to be one of the most efficient mechanisms to promote out-crossing [3]. SI is a widespread mechanism that has been reported in nearly one-half of angiosperm species [4]. However, SI has only been characterized in a few families and the underlying molecular and genetic factors that are involved in SI have only been described in detail in the Sporophytic Self-Incompatibility System (SSI) and in the Gametophytic Self-Incompatibility System (GSI) [5]. In those systems, self-incompatibility is genetically controlled by a multiallelic locus named *S* [6]. Following the discovery of sporophytic and gametophytic incompatibility [6], an array of other incompatibility systems, such as late-acting self-incompatibility [7] or cryptic self-incompatibility [8], have been reported in different families, although the cells and the mechanisms that are involved have not been identified. Interestingly, cryptic self-incompatibilty has been found in herkogamous species in which the growth of cross-pollen tubes is faster than that of self-pollen tubes [7], a pollen tube behavior that, to the best of our knowledge, has not been studied in GSI and should also be taken into account. However, this scenario has not been described in Rosaceae and it seems unlikely to occur in this family [9].

SSI has been mainly characterized in the Brassicaceae family [10]. In SSI, recognition factors are coded by the sporophyte and deposited extra-cellularly on the pollen grain wall. Pollen germination is arrested at the stigma surface when the *S*-allele of the haploid pollen matches either of the *S*-alleles of the diploid pistil [11]. GSI is the most abundant SI in angiosperms and it has been characterized in 18 families, including the Rosaceae [7]. GSI is controlled by a multiallelic locus *S*, which encodes the stylar and pollen *S*-determinants, and cell-cell recognition generally takes place in the style during pollen tube growth [12]. The stylar *S*-determinant, a ribonuclease (S-RNase), is a glycoprotein secreted into the style mucilage [13] that is composed of five highly conserved regions (C1–C5). Additionally, a single hypervariable region (RHV) is found in the S-RNase of Rosaceae, while two hypervariable regions (HVa and HVb) are observed in the S-RNase of Solanaceae and Plantaginaceae [14]. The pollen *S*-determinant is an F-box protein that contains two variable (V1 and V2) and two hypervariable (HVa and HVb) regions, and it is coded by pollen-specific F-box genes (*SFB*) [15]. 

Rosaceae constitutes the third most economically important family in temperate regions of the world, including several important crops, such as pear (*Pyrus communis*), apple (*Malus domestica*), sweet cherry (*Prunus avium*), almond (*Prunus dulcis*), and apricot (*Prunus armeniaca*) [16]. Most European common apricot cultivars have been traditionally considered as self-compatible [17]. However, with the purpose of introducing a source of resistance to sharka, some incompatible cultivars that were developed in North America have been used as parents in several breeding programs [18,19]. Sharka is the most important virus disease affecting *Prunus* in Europe and it has caused important economic damages in the last decades, becoming a limiting factor for apricot production in some areas. The release of these new breeding lines has resulted in the introduction of an increasing number of new apricot cultivars with unknown incompatibility information.

Self and intercompatibility relationships have been traditionally determined by performing field-controlled pollinations. The (in)compatibility is established by recording the percentage of fruit set four weeks after self- and cross-pollinations [20,21]. However, the efficiency of this method is conditioned by uncontrolled environmental factors [22]. Incompatibility can also be evaluated by observing pollen tube growth through the style in hand-pollinated flowers under the microscope. The stigma of apricot is papillate and wet, and the style shows a compact transmitting tissue enveloped by vascular bundles. Pollen grains germinate on the moist surface of the stigma within one day after pollination. The pollen tube penetrates into the stigma between the papillae, reaches the transmitting tissue and grows along the style in the following days. Pollen tube growth along the style takes 3–4 days [22]. However, pollen tube behavior can also be affected by environmental factors, mainly temperature. Thus, warm temperatures prior to flower opening result in lower fruit set, related to an asynchrony between the reproductive organs and the showy part of the flower [22]. Moreover, high temperatures can accelerate pollen tube growth kinetics [23]. Thus, in order to minimize environmental effects, the (in)compatibility phenotype can be evaluated in semi-in vivo culture of flowers in laboratory-controlled self- and cross-pollinations, followed by the subsequent observation of pollen tube behavior through the style under a fluorescence microscopy. This approach has been successfully used to determine self-(in)compatibily and incompatibility relationships between cultivar, in apricot [24] and other *Prunus* species [25].

Moreover, since the first isolation of a partial cDNA encoding an *S*-locus specific glycoprotein from *Brassica oleracea* [26], the development of molecular techniques based on the sequence of the *S*-locus has allowed for the identification of self-(in)compatibility and the incompatibility relationships among cultivars in numerous species. In *Prunus*, the sequence of the *S-RNase* revealed two introns with considerable length polymorphism that have been used to distinguish among *S*-haplotypes [27,28,29,30,31]. Thirty-three alleles have been reported so far in apricot (*S*_1_ to *S*_20_, *S*_22_ to *S*_30_, *S*_52_, *S*_53_, *S_v_*, *S_x_*), including one allele for self-compatibility (*S_c_*) [32,33,34,35,36]. Additional alleles have been included in the NCBI database but not yet published showing misidentification and the appearance of numerous homologies [24,35]. *S-*genotyping has allowed for classifying the cultivars in their corresponding incompatibility group according to their compatibility relationships. Self-incompatibility is considered when the genotype of pollen matches with one of the *S-*alleles of the pistil. Thus, those self-incompatible cultivars with the same *S*-alleles are inter-incompatible and are allocated in the same incompatibility group, whereas cultivars from different groups with at least one different *S*-allele are inter-compatible [35]. In apricot, 23 incompatibility groups have been stablished [24,37,38,39,40], which provides useful information to apricot growers and breeders.

To fill the lack of knowledge on self-(in)compatibility and incompatibility relationships in the increasing number of new apricot cultivars, in this work we identified the *S*-genotype of 44 apricot cultivars and selections by PCR analysis, and classified them according to their *S-RNase* alleles in nine incompatibility groups. Of those, the *S*-genotype of 43 is reported here for the first time. Additionally, in those cases in which the *S*-genotype could not be identified by molecular approaches, self-incompatibility was established by the observation of pollen tube behavior under the microscope following self-pollination. Our results elucidated incompatibility relationships among a high number of apricot cultivars with previous unknown pollination requirements providing valuable information to design new crosses in apricot breeding programs and selecting self-compatible and inter-compatible varieties in new apricot orchards.

## 2. Results

**Figure 1:**
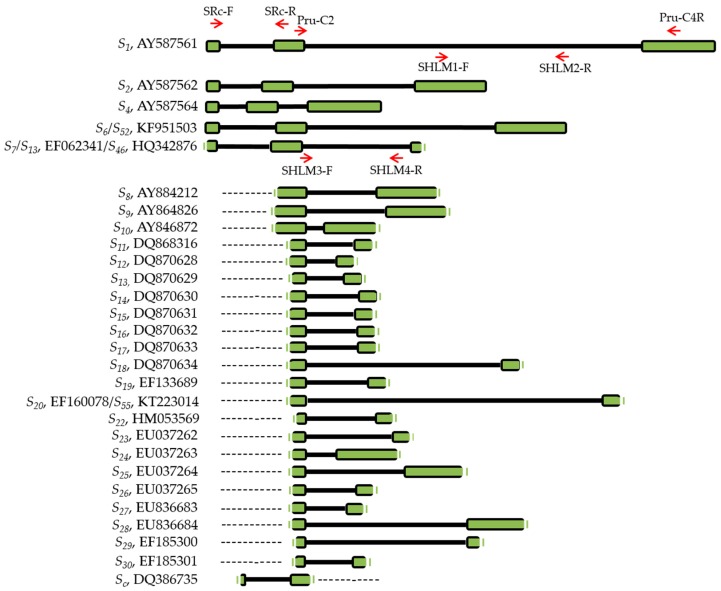
Gene structure of the *S*-alleles sequenced, showing the exons in green square and including the primers used in this study for the alleles *S*_1_ and *S*_7_/*S*_13_/*S*_46_. Unknown sequences are represented by dotted lines. The first column shows *S*-genotype and gen bank accession. *S*_1_ [41], *S*_2_ [41], *S*_3_ [41], *S*_4_ [41], *S*_6_/*S*_52_, *S*_7_/*S*_13_ and *S*_46_ [Unpublished], *S*_8_ [42,43], *S*_9_ [42], *S*_10_ [42], *S*_11_ [34], *S*_12_ [34], *S*_13_ [34], *S*_14_ [34], *S*_15_ [34], *S*_16_ [34], *S*_17_ [34], *S*_18_ [34], *S*_19_ [34], *S*_20_ [34]/*S*_55_ [Unpublished], *S*_22_ [44], *S*_23_ [44], *S*_24_ [44], *S*_25_ [44], *S*_26_ [44], *S*_27_ [44], *S*_28_ [44], *S*_29_ [44], *S*_30_ [44], *S_c_* [43].

Self-compatibility of 44 cultivars and selections (Table 1) was evaluated by *S-RNase* allele identification using PCR analysis, amplifying the conserved regions of the apricot *S-RNase* locus. We first identified the *S-RNase* alleles using the primers SRc-F/SRc-R for the amplification of the first intron (Table 1 and Table 2, Figure 1). Because some *S-RNase* alleles, such as *S*_1_ and *S*_7_ or *S*_6_ and *S*_9_ showed amplified fragments of similar size of the first intron in 27 genotypes, we also used the primers Pru-C2 and PruC4R that amplify the second *S-RNase* intron. These additional primers enabled identifying the *S*-allele of 13 of those 27 genotypes. The primers Pru-C2 and PruC4R also amplified a fragment of 2 kb in the genotype ‘T007’. This fragment size in the second intron has not been previously reported in other apricot cultivars. Thus, we further analyzed this PCR fragment by cloning and sequencing (Figure S1). We obtained a sequence of 2002 bp that showed a 99% of identity with the *S*_20_ allele (EF160078, [34]) and the unpublished *S*_55_ allele (KT223014). Thus, we identified one of the alleles of the *S*-genotype of ‘T007’ as *S*_20_.

**Figure 2:**
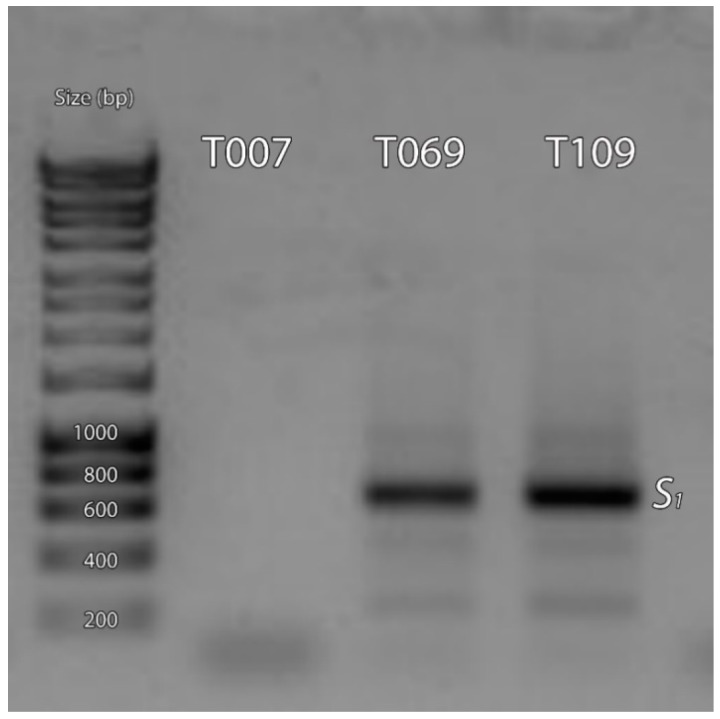
PCR amplification of the *RNase* second intron region using the specific primers SHLM1 and SHLM2 for the identification of the *S*_1_ allele in T007 (*S*_20_), T069 (*S*_1_*S*_2_), and T109 (*S*_1_).

The combination of the primers Pru-C2 and PruC4R was inefficient in 15 genotypes with no amplification fragments obtained. Therefore, new specific primers were designed to amplify the second intron of the *S*_1_ and *S*_7_ alleles (Table 2, Figure 1). The primers SHLM1 and SHLM2 amplified a fragment of 650 bp from the *S*_1_ allele (Figure 1 and Figure 2). However, while the sequence of *S*_1_ has been previously reported [41] (AY587561), the *S*_7_ allele has only been identified by PCR analysis and no sequence has been yet included in the NCBI database. Thus, using the primers Pru-C2 and PruC4R, we cloned and sequenced the fragment of 900 bp that was obtained from the cultivar ‘Charisma’ (*S_c_*/*S*_7_, [24]), which corresponds to the *S*_7_ allele [33]. Surprisingly, the cloned sequence resulted in a fragment of 915 bp (Figure S2) that showed a 99% identity with the partial coding sequence (790 bp, EF062341, unpublished), of the *S*_13_ allele and with a fragment of 830 bp that corresponds to the unpublished *S*_46_ allele (HQ342876). The *S*_13_ allele (DQ870629) has also been identified by Zhang et al. [34], but the sequence of the gen bank accessions EF062341 and DQ870629 are different, and, consequently, both sequences have been considered as two different *S*-alleles. Our results suggest that *S*_7_, *S*_13_ (EF062341), and *S*_46_ could indeed be the same allele. Based on the sequence of 915 bp, we designed the primers SHLM3 and SHLM4 that amplified a fragment of 413 bp. The primers Pru-C2/PruC4R, SHLM1/SHLM2, and SHLM3/SHLM4 enabled the identification of the *S* genotype of 27 additional cultivars and selections (Table 1).

**Figure 3:**
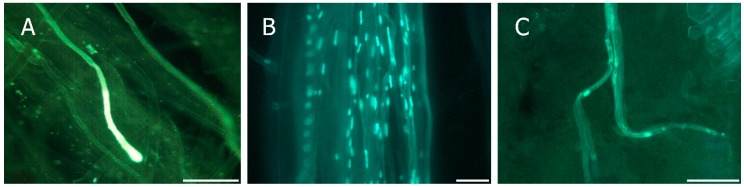
Pollen tube growth in apricot pistils. (**A**) Pollen tube arrested in the upper half of the style in a self-incompatible cultivar; (**B**) Pollen tubes growing along the style; and, (**C**) Pollen tubes at the base of the style in a compatible cross. Scale bars, 100 µm.

Due to the identical sequence of the *RNase* gene in the *S_c_* and *S*_8_ haplotypes [43], the pairs of primers SRc-F/SRc-R and Pru-C2/PruC4R could not be used for distinguishing between those two alleles. Therefore, the *S_c_* and *S*_8_ alleles can only be identified using the primers AprFBC8-F and AprFBC8-R that amplify a fragment of approximately 500 bp in the *S_c_* allele and 150 bp in the *S*_8_ allele from the V2 and HVb variable regions of the *SFB* gene [39]. Thus, the *S*-genotype of 13 cultivars was identified using the primers AprFBC8-F and AprFBC8-R (Table 2). Moreover, self-(in)compatibility was also evaluated by direct observation of pollen tube growth in the pistil after self-pollination in five selections (“C003”, “C007”, “C009”, “C012”, and “C014”), in which the primers AprFBC8-F/AprFBC8-R were not able to differentiate between the *S_c_* and *S*_8_ alleles. Pollen germination was observed on the stigma in all cultivars. Pollen tube growth was arrested in the upper half of the style, showing a terminal callose plug and end thickening (Figure 3A) in three genotypes (“C007”, “C009”, and “C012”) that were considered as self-incompatible. Self-compatibility was considered in two genotypes (“C003” and “C014”), in which the pollen tubes grew along the style (Figure 3B) and at least one of them reached the base of the style in most of the pistils that were analyzed (Figure 3C). As expected, the pollen used was viable and all cross-pollinated pistils displayed pollen tubes at the base of the style (Table 3). 

## 3. Discussion

The *S*-allele composition of 44 apricot genotypes, including new cultivars and advanced selections of three breeding programs, of which 43 have been reported here for the first time, has been identified. *S*-genotyping by PCR analysis allowed for the identification of the two *S*-alleles in 21 genotypes. Approximately half of the genotypes analyzed (55%) were self-compatible, including commercial cultivars, such as “Murciana” or “Dorada” [45,46]. The remaining genotypes, mostly advanced breeding selections, were considered as self-incompatible. This *S-*allele identification can speed up breeding programs by selecting self-compatible genotypes at the seedling stage or genotypes with the desired *S*-allele composition. The results confirm the tendency of the increase in the number of self-incompatibility genotypes in apricot that is in contrast with the previous scenario, when most European cultivars were self-compatible [47]. 

According to their *S*-allele composition, 13 self-incompatible cultivars have been allocated in nine previously determined incompatibility groups [24,37,38,39,40]. “Primaya” (*S*_1_*S*_6_), “A106” (*S*_1_*S*_9_), and “C009” (*S*_6_*S*_8_) have been assigned to XXIV-XXVI groups, three new incompatibility groups that have been reported herein for the first time. By contrast, 15 cultivars had the *S_c_* allele and they were characterized as self-compatible, and, consequently, can be considered as universal pollen donors. Results herein confirm the *S-*alleles of ‘Beliana’ (*S_c_S*_7_) [48]

The PCR fragment of the first intron of *S*_8_ was identical to *S_c_*. Moreover, both S-RNases showed equal isoelectric points [43], suggesting that the S-RNase of *S*_8_ and *S_c_* alleles are identical. Thus, it is likely that *S_c_* could derive from the *S*_8_ allele [43]. In this case, as it has also been reported in other *Prunus* species [25,49], the breakdown of the incompatibility system can be related to a mutation outside of the *S*-locus [50]. Indeed, an insertion of 358 bp in the *SFB* gene causes a loss of the incompatibility [50] that was also observed in the *SFB_c_* gene but not in the sequence of *SFB*_8_ [43]. That is why primers have been designed based on the *SFB* sequence for the identification of *S_c_* and *S*_8_, such as the primer pair AprFBC8-R/F. Thus, using these primers, we could distinguish between *S_c_* and *S*_8_ in 13 apricot genotypes. However, *S_c_* and *S*_8_ could not be distinguished in five genotypes. Although the PCR-based method has been a preferred technique for *S*-allele identification, mismatching of PCR primers can result in no amplification. This might be the case in these five selections, in which an amplified fragment was not obtained using the primer pair AprFBC8-R/F. As an alternative, we performed controlled pollinations that were carried out in the laboratory and pollen tube growth was observed under the microscopy to determine self-(in)compatibility. The results showed that two genotypes behaved as self-compatible and they were assigned with allele *S_c_*, whereas the other three were self-incompatible and were assigned with allele *S*_8_.

The inaccuracy of the PCR-based method could also be reflected in the amplification of just one allele. Indeed, 23 of the 44 genotypes that were analyzed in this work show only one *S*-allele after PCR amplification. This could be due either to homozygosis in the case of the *S_c_* allele or with amplification problems in the other genotypes. Thus, the *S*-genotype of “Dorada”, “Memphis”, “Milord”, “Murciana”, “Oscar”, “Sherpa”, and “T003”is likely to be *S_c_S_c_*. Further sequencing work is under way to identify the other *S* allele in the remaining 16 genotypes. *S*-allele analysis by band identification in a gel can also provide inaccurate identification. It also should be taken into account that the *S-RNase* sequence has only been revealed in some *S*-alleles, and, in most of the cases, such as *S*_9_*, S*_15_, or *S*_27_, it has been only partially sequenced, and/or not including introns that are essential for fragment sizing in *S*-allele identification. The use of an automatic fragment analyzing system can also report small differences in fragment size; for example, in *S*_2_ allele (327 bp [33]; 334 bp [24]; 332 bp [51]) or *S_c_* (353 bp [33]; 358 bp [24]; 355 bp [51]). Taken together, all this has led to numerous homologies [2,4,35]. That is the case of *S*_6_ and *S*_52_ [24] or *S*_20_ and *S*_55_, and *S*_7_, *S*_13_ (EF062341) and *S*_46_ shown in this study.

There is a need to standardize the criteria for the *S-*allele identification in different laboratories, such as the use of the same primer pairs and the complete sequencing of the *S*-alleles, including introns. This will facilitate *S*-allele identification avoiding confusion and it will provide valuable information for apricot growers and breeders. Results herein allow for establishing incompatibility relationships among apricot cultivars with previous unknown pollination requirements, allowing the selection of the appropriate parents in the design of new crosses in apricot breeding programs and self-compatible and inter-compatible cultivars in new commercial orchards. Moreover, the approach that is followed in this work can be of interest to other fruit crops in the Rosaceae with similar problems to those faced by apricot.

## 4. Materials and Methods

### 4.1. Plant Material

Plant material was collected from different apricot germplasm collections and orchards of Aragon, Cataluña, and Extremadura (Spain). Young leaves from 16 new apricot cultivars and 28 advanced selections from different breeding programs were used in this study for PCR analysis (Table 1). Moreover, flowers were collected from five of the selections (“C003”, “C007”, “C009”, “C012”, and “C014”) for pollination experiments (Table 3).

### 4.2. DNA Extraction

Young leaves from the 44 cultivars and selections were collected for the identification of self-incompatibility alleles. Genomic DNA was extracted following the protocol described by Hormaza [52] and using a DNeasy Plant Mini Kit (Qiagen, Hilden, Germany). The DNA quantification was performed by using a NanoDrop™ ND-1000 spectrophotometer (Bio-Science, Budapest, Hungary).

### 4.3. S-RNase Allele Identification by PCR Analysis

Self-incompatibility was stablished by *S*-allele identification through PCR amplification of *RNase* and *SFB* regions. Fluorescently labelled forward primer SRc-F with the reverse primer SRc-R [33,41] were used to amplified the first intron region of the *S-RNase* gene. PCR amplifications were carried out in 15 µL reaction volumes, containing 10× NH_4_ Reaction Buffer, 25 mM Cl_2_Mg, 2.5 mM of each dNTP, 10 µM of each primer, 100 ng of genomic DNA, and 0.5 U of BioTaq^TM^ DNA polymerase (Bioline, London, UK). The temperature profile used had an initial step of 3 min at 94 °C, 35 cycles of 1 min at 94 °C, 1 min at 55 °C and 3 min at 72 °C, and a final step of 5 min at 72 °C. *S_c_* and *S*_8_ alleles had an identical fragment length of 355 bp from the first intron, amplified using the primers SRc-(F/R) [42,43]. Thus, the SFB specific primers, AprFBC8-F and AprFBC8-R, were used to distinguish between both alleles. The identification was carried out according to the PCR conditions of Halasz et al. [39]. The sizes of the products that were obtained by PCR were analyzed in a CEQ^TM^ 8000 capillary electrophoresis DNA analysis system (Beckman Coulter, Fullerton, CA, USA) and compared and classified according to Vilanova et al. [33] and Kodad et al. [51]. For the amplification of the *RNase* second intron region, the primers Pru-C2 and Pru-C4R were used, as recommended by Vilanova et al. [33], but with the addition of 10 cycles and using 55 °C of annealing temperature, as indicated by Sonneveld et al. [53]. The amplified fragments were separated on 1% (*w*/*v*) agarose gels and DNA bands were visualized using the nucleic acid stain SYBR Green (Thermo Scientific, St Leon-Rot, Germany).

Since the PruC2/PruC4R primer combination was inefficient to distinguish *S*_1_ and *S*_7_ alleles, new specific primers were designed from the second intron, SHLM1 and SHLM2 for the identification of the *S*_1_-haplotype and SHLM3 and SHLM4 for the identification of the *S*_7_ haplotype (Table 2, Figure 1). For *S*_1_ identification, *Taq* DNA polymerase (Qiagen, Hilden, Germany) was used with a temperature profile of an initial step of 94 °C for 2 min, 35 cycles of 94 °C for 30 s, 62 °C for 1 min and 30 s and 72 °C for 2 min, and a final extension of 72 °C for 5 min. For *S*_7_ identification, the amplification was performed using Phusion^®^ High-Fidelity DNA Polymerase (Thermo Scientific, St Leon-Rot, Germany) with the following temperature profile: 30 s at 98 °C, 35 cycles of 10 s at 98 °C, 30 s at 51 °C, and 1 min at 72 °C, with a 5 min final extension step at 72 °C.

### 4.4. Sequencing of Genomic PCR Products

In order to design *S*_7_-specific primers, a fragment of 900 bp obtained by using the primers PruC2/PruC4R was isolated using the NucleoSpin Gel and PCR Clean-up (Macherey-Nagel, Düren, Germany). Cloning was performed using CloneJET PCR Cloning Kit (Thermo Scientific, St Leon-Rot, Germany) and by electroporation in *E. coli* Single-Use JM109 Competent Cells (Promega Biotech Ibérica SL, Madrid, Spain). The search for similarities in the sequences of the NCBI database was performed with BLAST (http://www.ncbi.nlm.nih.gov/BLAST, version 2.2.10).

### 4.5. Pollination Experiments

Pollination crosses were carried out with the five selections in which the identification of the *S*_8_ and *S_c_* alleles was not possible by PCR amplification. To establish self-(in)compatibility, self-pollinations and cross-pollinations were carried out in semi-in vivo culture of flowers in the laboratory and pollen tube growth was observed under fluorescence microscopy. As control, flowers of each cultivar were pollinated with pollen from the cultivar “Katy”, which is known as the universal pollinizer for apricot (Table 3) [54].

**Figure 4:**
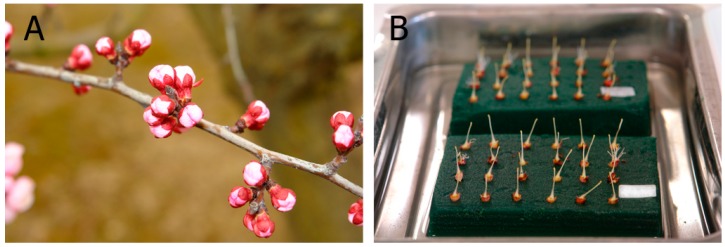
Apricot flowers at balloon stage in the field (**A**) and pistils on florist foam in water in the laboratory (**B**).

For each self- and cross-pollination, a group of 20–25 flowers was collected from the trees at the balloon stage (Figure 4A), corresponding to stage 58 on the BBCH scale for apricot [55]. Flowers were emasculated in the laboratory to avoid self-pollination, placed on florist foam in water (Figure 4B) at laboratory temperature, and hand pollinated with the help of a paintbrush 24 h after emasculation. Pollen was obtained from flowers at the same balloon stage by removing and drying the anthers at laboratory temperature during 24 h. Pollen grains were sieved by using a fine mesh (0.26 mm) and then used immediately or frozen at −20 °C until further use at laboratory temperature [56]. After 72 h, when the pollen tubes had enough time to reach the ovary [57], pistils were fixed in ethanol (95%)/acetic acid (3:1, *v*/*v*) during 24 h, and conserved at 4 °C in 75% ethanol [57]. Pollen viability was also evaluated after each pollination. Pollen was scattered on a solidified pollen germination medium [58] and observed under the microscope 24 h after pollen germination. Pollen grains were considered to be viable when the length of the growing pollen tube was higher than the pollen grain diameter.

For histochemical preparations, the pistils were washed three times for 1 h with distilled water and left in 5% sodium sulphite at 4 °C. After 24 h, they were autoclaved at 1 kg/cm^2^ during 10 min in sodium sulphite [59] to soften the tissues, squashed, and stained with 0.1% (*v*/*v*) aniline blue in 0.1 N K_3_PO_4_ [60] to stain callose deposition during pollen tube growth under the microscope [21,22,24,56,61]. Pollen tube growth along the style was observed by a Leica DM2500 microscope (Cambridge, UK) with UV epifluorescence using 340–380 bandpass and 425 longpass filters.

Pollen tube growth was recorded on 8–18 pistils in each self-pollination and at least 4–18 pistils in control crosses. Cultivars were considered as self-compatible when most of the pistils displayed at least one pollen tube reaching the base of the style. Self-incompatibility was considered in self-pollinated flowers when pollen tube growth was arrested along the style in all pistils.

## Figures and Tables

**Table 1:** Incompatibility groups and *S-RNase* genotype of the 44 apricot cultivars and selections analyzed in this study.

Incompatibility Group	*S-RNase* Genotype	Apricot Genotypes Analyzed in this Study
I	*S*_1_ *S*_2_	T069
		T120
		T139A
II	*S*_8_ *S*_9_	C007 ^1^
V	*S*_2_ *S*_8_	C012 ^1^
VIII	*S*_6_ *S*_9_	Cheyenne
		T001
XVIII	*S*_1_ *S*_3_	A150
XXII	*S*_3_ *S*_9_	A153
		Kosmos
XXIV ^2^	*S*_1_ *S*_6_	Primaya
XXV ^2^	*S*_1_ *S*_9_	A106
XXVI ^2^	*S*_6_ *S*_8_	C009 ^1^
Self-compatible cultivars	*S*_2_ *S_c_*	Kalao
		Regibus
	*S*_3_ *S_c_*	C014 ^1^
		Rambo
	*S*_4_ *S_c_*	T002
	*S*_7_ *S_c_*	Beliana
	*S*_9_ *S_c_*	C003 ^1^
		Lido
	*S_c_*	Dorada
		Memphis
		Milord
		Murciana
		Oscar
		Sherpa
		T003
	*S*_1_	A154
		T004
		T005
		T109
		T124
		T139B
		T140
	*S*_2_	Cyrano
		T098
	*S*_3_	Mikado
		T006
	*S*_7_	T116
	*S*_9_	A151
		A152
		A155
	*S*_20_	T007

^1^*S_c_*/*S*_8_ allele identified using fluorescence microscopy; ^2^ Incompatibility groups first reported in this study.

**Table 2:** Primers used in this study for the identification of *S*-alleles in *Prunus armeniaca*.

Primers	Amplified Region	Sequence (5′ → 3′)	Reference
SRc-F	*S-RNase* 1st intron	CTCGCTTTCCTTGTTCTTGC	[41]
SRc-R	*S-RNase* 1st intron	GGCCATTGTTGCACCCCTTG	[41]
Pru-C2	*S-RNase* 2nd intron	CTTTGGCCAAGTAATTATTCAAACC	[27]
Pru-C4R	*S-RNase* 2nd intron	GGATGTGGTACGATTGAAGCG	[27]
AprFBC8-F	*SFB*	CATGGAAAAAGCTGACTTATGG	[39]
AprFBC8-R	*SFB*	GCCTCTAATGTCATCTACTCTTAG	[39]
SHLM1-F ^1^	*S*_1_-*RNase* 2nd intron	GGTGGAGGTGATAAGGTAGCC	
SHLM2-R ^1^	*S*_1_-*RNase* 2nd intron	GGCTGCATAAGGAAGCTGTAGG	
SHLM3-F ^1^	*S*_7_-*RNa*se 2nd intron	TATATCTTACTCTTTGGC	
SHLM4-R ^1^	*S*_7_-*RNase* 2nd intron	CACTATGATAATGTGTATG	

^1^ Specific primers designed in this study.

**Table 3:** Pollen tube behavior in the pistils of five apricot selections after self- and cross-pollinations.

Cultivar	Number of Pistils Examined	Pistils (%) with Pollen Tubes			Percentage of Style Travelled by the Longest Pollen Tube	Mean Number of Pollen Tubes at the Base of the Style	Compatible (C) or Incompatible (I) Behavior
		in the Middle of the Style	at the Base of the Style	Reaching the Ovule			
**Self-pollination**							
C003	10	100	90	80	100	2	C
C014	18	100	94	83	100	2.1	C
C007	8	75	0	0	57	0	I
C009	10	60	0	0	56	0	I
C012	9	100	0	0	66	0	I
**Cross-pollination**							
C003 × Katy	18	100	78	78	100	1	C
C014 × Katy	5	100	100	100	100	3.4	C
C007 × Katy	5	100	80	80	100	1	C
C009 × Katy	5	100	80	80	100	1.6	C
C012 × Katy	4	100	100	100	100	1.5	C

## Supplementary Materials

Supplementary materials can be found at http://www.mdpi.com/1422-0067/19/11/3612/s1.

## Abbreviations

GSI	Gametophytic Self-Incompatibility System
SSI	Self-Incompatibility System
SI	Self-Incompatibility
