# Supplementary material for: Identification of Self-Incompatibility Alleles by Specific PCR Analysis and S-RNase Sequencing in Apricot
Source: Int J Mol Sci. 2018 Nov 15;19(11):3612. doi: 10.3390/ijms19113612 (PMC6274852; doi:10.3390/ijms19113612)
Supplement: Supplementary file 1 [file ijms-19-03612-s001.pdf]

Figure S1. S_{20}/S_{55} allele. Sequence of 2002 bp obtained from the the genotype 'T007 using the primers Pru-C2 and PruC4R. Predicted exons and intron are showed in red and black respectively.

CATATCAATTAGACAGCGCATTAGTTGAATGAGGGCGAATTTAGGACGGGAAAGTGGTATGTATTGTT
TCAAATATTTTTTTTTCTTTCATATTGCTCTTAGAAAATTAGATTGTCATATGAAGATAATACATTTTATTT
AATAAGCCATGGTCTTGGATAAAATTTTGATGTTTGTCCTCTGCTAGGCACATTATTTTGAATCTCTTTTG
AAATAGGGGAAATATATGGCTAAGTACATTTATATTCATAGGTATTGATGTATATGTCAAGTCAGAATC
GTGGGAAATAAAAAAAACACACATAATTTGACACACTTTTTAATCTTGAGATGAATCACATTGAAAATC
ATTTACCATTTAAGAGAAATGCATTTCTTTTATTACTTCATTGAAAACCCAAATACATTTAATTATGTATA
CTCTCTCACTTCCCTTTACCTATTATCATTTTATTCAAAAGCAACCTCCTTTATTACTCACCACTTAAGAGT
ATTCTGACTCTCTAAACCACCTTATTAGATAAAATTTAAAAAGGAAGTGAGAAAACTCATCTCTAACCAT
GCTTTTTATCCAGCTCATAGGGAGACCTTTACGAGCTTTTAAATCTGAGGAGAGAGAATGGACCCCTAA
TGGCTCCATATAATATATTGGTGCCTTATTTAATGAATATTTTAAACCTTTAATTAATATTGCCTATTTTTT
ATATAGAGATGGACCATTTATGTTGAATTAAAAAAGAATTATAACATTTATGACTCCCTAAAGAATATG
ATGGAAGTTGAAATTTTATAGATAGCTCCTAAAATAGATTTTGTGTGTTTTAGCTAAATTTTAACTACAA
AAATAGAAAGAATGATTCCATGTGCATAATCATAATACTTGACACCTATCCACCATCCTTAATACTTGAT
ACGTGTCCATATGCATAATCATGGTTTCATATACAAAATAAAAAGTTAATCATGGTTATTCATATGAACA
CATGTCAAGTGTTGAAAATAATAATGAGGATATACACTGGGTTAAAAAGGTGAGTAAAAAATGCTCAC
CTTTCTTCATTGTCTTCCCTTCCCCTGTGCCATGGGTTAGCCATAGTCTCTTCATCCCCAACGTTCCCTTCT
ACCTTACCACCCATACTCCCCCTTCGCTACATTATTCCTCTTAAAAATATTTTTCTCTTAAAACCCTTCAGA
ACCCATAAAAATAAAAAATAAAAAAAAGAACCCCAAATGCACACCCCTCTCCCCGGTAGGACCAGGGT
CCACATCACACCGCGTTTCTGCACCACCACCAGATCTACCATTTTTAGGATCCTACATTATTGGCTTGTGT
GGTAACACAGCCACTTGATCCATCTTACTAGACCCATTGGAGCTACCGCATGTCTAGCTCCACCAAGAG
AGAGTAACAGGTAGGAAACAGTATAAAGGAGGAGAGAAAAAATAAGGGACATTTTGAAAAAAGCAA
AAGTATATAAATTTTTTTATATGTTTCTAAGAAGTAAAAAAAACATGTTAATTTTTTTATTTAAAAAATAC
TCATGACAGATACATTTTGAGGCATTAAAAATGTGTATATAAATTTTTGGTTTTTTGTGCTCATCGATCCT
GATCTATTTTTATAGTAATGGATATGATCATCTAATTAAAGTACCTACCATTTTGTGCTTGTTTATTCAAA
ATATTGTACCTAATGAAAGAATGAAAATGGTAATAACAATCTTATACAAAAATGAAACTCTAACTATCCC
TTACGTTTTTACTTTTTCTCCAATTATGTATATTTTGCTTGGATGTCTCAGTACCCTCAGTTGCGAACCAA
ACTGAAGAAATCTTGGCCGGACGTGGAAAGTGGGAATGATACAAAATTTTGGGAAGGCGAATGGAAC
AAACATGGTACATGTTCTGAAGAGAAACTAAACCAAATGCAATACTTCGAGAGATCCCACAACATGTG
GAGGTCGTACAATATTACAGAGATCCTTAAAAACGCTTCAATCAA

Figure S2. S_7, S_{13} and S_{46} allele. Sequence of 915 bp obtained from the cultivar 'Charisma' using the primers Pru-C2 and PruC4R. Predicted exons and intron are showed in red and black respectively.

CTTTGGCCAAGTAATTATTCAAACCCAAGGAAGCCTAGTAATTGCAATGGGTCACAATTTGACGCAAGG
AAAGTGGTACGTATTGTTTCATTATTTTATATCTTACTCTTTGGCATTTAGTTTTTTAGGTTTTTTTATATA
TAGGAATTAGTGTTTAGAAAATTAGATTGTCATGTGAAGATTTTAATAAATAAATAAACCTTTTTCAATA
AGCCTTGGGTGTTATAGATTAAATTTTGATGTTGGTTCTTAGTTAGACACATTATTTTGAATATATAGTT
AAGTACAAAATGGCAAGTACATATTAATATACTTTCGAAAATATAATGGATCTGCTCATCTAATACCATT
TTGTACTAATGCATATATGCAAAACATTGTACATCAAATTCTTTTTAAAGCAAGGCTATAATATATTATTG

GAGATTAAACTCAAAATTAATGCTCGGATTTAATGAGACAAAAAAACAATCTTTGTATTTTTAGTACAAG
CGACAATATAAATTACATGAGAATGAGTTTCTCACATACACATTATCATAGTGTCATAGAAGTTGGAATT
TAAGACTAGAGATCTACAAATCAAGACTCTTTTTATATTGGGCTAGACTCCGTTAACGACAAAAACTCA
ATAATATTCAAGAATGAAAATCTAATTATCATTTATTCGATTTACTTTTTCTCAAATATGTGTCTACATTGT
TTGGATGTCCCAGTCCCCTCAATTGCGATCAAAACTGAAGATATCTTGGCCCGACGTGGAAGGTGGCAA
TGATACACAATTTTGGGAAGGCGAATGGAACAAACATGGTACTTGTTCCGAAGAGACACTTGACCAAA
CGCAATACTTCGCGCGATCCCACGCGTTTTGGAACATGCGCAATATTACGGAGGTCCTTAAAAACGCTT
CAATCG
